# Cost-effectiveness and impact of pre-exposure prophylaxis to prevent HIV among men who have sex with men in Asia: A modelling study

**DOI:** 10.1371/journal.pone.0268240

**Published:** 2022-05-26

**Authors:** Debra C. ten Brink, Rowan Martin-Hughes, Mark E. Minnery, Aaron J. Osborne, Heather-Marie A. Schmidt, Shona Dalal, Kimberly E. Green, Reshmie Ramaurtarsing, David P. Wilson, Sherrie L. Kelly

**Affiliations:** 1 Burnet Institute, Melbourne, Victoria, Australia; 2 United Nations Programme on HIV/AIDS, Regional Office for Asia and the Pacific, Bangkok, Thailand; 3 World Health Organization, Geneva, Switzerland; 4 PATH, Hanoi, Vietnam; 5 Institute of HIV Research and Innovation, Bangkok, Thailand; 6 Monash University, Melbourne, Victoria, Australia; 7 Bill and Melinda Gates Foundation, Seattle, Washington, United States of America; HIV/STI Surveillance Research Center and WHO Collaborating Center for HIV Surveillance, Institute for Future Studies in Health, Kerman University of Medical Sciences, ISLAMIC REPUBLIC OF IRAN

## Abstract

**Introduction:**

More than 70% of new HIV infections in Asia occurred in eight countries in 2020: Cambodia, China, India, Indonesia, Myanmar, Nepal, Thailand, and Vietnam—with a rising incidence among men who have sex with men (MSM). The World Health Organization (WHO) recommends pre-exposure prophylaxis (PrEP) for those at risk of acquiring HIV, yet wide-scale implementation of PrEP, on a daily or event-driven basis, has been limited in Asia.

**Methods:**

The Optima HIV model was applied to examine the impact of scaling-up PrEP over five-years to cover an additional 15% of MSM compared with baseline coverage, a target deemed feasible by regional experts. Based on behavioral survey data, we assume that covering 15% of higher-risk MSM will cover 30% of all sexual acts in this group. Scenarios to compare the impact of generic-brand daily dosing of PrEP with generic event-driven dosing (15 days a month) were modelled from the start of 2022 to the end of 2026. Cost-effectiveness of generic versus branded PrEP was also assessed for China, the only country with an active patent for branded, higher cost PrEP. The impact on new HIV infections among the entire population and cost per HIV-related disability-adjusted life year (DALY) averted were estimated from the beginning of 2022 to the end of 2031 and from 2022 to 2051.

**Results:**

If PrEP were scaled-up to cover an additional 15% of MSM engaging in higher-risk behavior from the beginning of 2022 to the end of 2026 in the eight Asian countries considered, an additional 100,000 (66,000–130,000) HIV infections (17%) and 300,000 (198,000–390,000) HIV-related DALYs (3%) could be averted over the 2022 to 2031 period. The estimated cost per HIV-related DALY averted from 2022 to 2031 ranged from US$600 for event-driven generic PrEP in Indonesia to US$34,400 for daily branded PrEP in Thailand. Over a longer timeframe from 2022 to 2051, the cost per HIV-related DALY averted could be reduced to US$100–US$12,700.

**Conclusion:**

PrEP is a critical tool to further reduce HIV incidence in highly concentrated epidemics. Implementing PrEP in Asia may be cost-effective in settings with increasing HIV prevalence among MSM and if PrEP drug costs can be reduced, PrEP could be more cost-effective over longer timeframes.

## Introduction

The use of pre-exposure antiretroviral drugs as an additional option to prevent HIV acquisition has been recommended by the World Health Organization (WHO) for those at “substantial risk” of HIV infection, referring to populations where HIV incidence is greater than three per 100 person-years of life [1].

In 2020, 16% of all new HIV infections worldwide were estimated to have occurred in Asia [2]. Together, Cambodia, China, India, Indonesia, Myanmar, Nepal, Thailand, and Vietnam accounted for an estimated 70% of all new HIV infections in Asia in 2020 [2–4]. These infections were predominantly concentrated among key populations, with the UNAIDS Global 2021 Report outlining a concerning increase among men who have sex with men (MSM) [2, 5]. MSM accounted for almost 53% of new HIV infections in Asia among 15- to 49-year-olds in 2020 [2]. As condom use remains low in this population, other prevention mechanisms such as PrEP may provide a more accessible prevention option for MSM. Other regions with notable HIV incidence among MSM, such as Australia, Europe, and the United States, have shown significant reductions in new HIV infections associated with PrEP [6].

Amongst the eight countries in Asia, only Thailand and Vietnam have thus far rolled out large-scale PrEP programs for key populations [5]. In 2016, Thailand offered PrEP for MSM and transgender women, the first country in the region to do so [7], with Vietnam following suit in 2017 [8]. Cambodia began a phased approach to national rollout of PrEP in 2019 [9]. China, India, Myanmar, and Nepal have implemented PrEP on a demonstrative basis from 2019 onwards [2], with a multicenter trial ongoing in China since 2018 [10]. Estimates from AIDS Vaccine Advocacy Coalition data (AVAC) PrEPWatch indicate that among these eight countries, Thailand has the largest number of people on PrEP, with an estimated 41,000 current users as of 27 January 2022, an estimated number of 29,000 in Vietnam, 4,500 in China, 3,000 in Nepal, and 1,500 in India [11]. The estimates for Cambodia, Indonesia, and Myanmar were less than 500 (UNAIDS and WHO communication), suggesting a limited number of PrEP users to date.

The most widely implemented PrEP drug regimens are a daily pill in an oral combination of tenofovir disoproxil fumarate (TDF) (300mg) with emtricitabine (FTC) (200mg) or lamivudine (3TC) (300mg) tablets [12, 13]. In 2019, WHO recommended an additional option to the daily PrEP regimen, namely event-driven or “2+1+1” dosing for PrEP [14]. Findings from ongoing studies in France and Canada report that event-driven dosing, two tablets 2 to 24 hours before, followed by one tablet 24 hours after and another 48 hours after a single high-risk event (e.g., condomless anal sex), prevent HIV infections to the same degree as daily PrEP for MSM [15].

High drug costs for generic as well as branded PrEP, have been cited as a key determinant for the cost-effectiveness of PrEP [16–21]. Encouragingly, prices have decreased markedly over the last few years with increasing numbers of pre-qualified generic versions of TDF/FTC entering the market, providing an opportunity for countries to acquire PrEP at much lower cost [22]. Nevertheless, existing literature finds that PrEP may be limited in its cost-effectiveness, but may be more cost-effective when targeting populations with higher incidence of HIV. A modelling study examining the impact of PrEP in a theoretical hyperendemic setting determined that a delivery cost of PrEP of US$250 per person per year was too high for implementation and scale-up to be cost-effective, thus limiting any added potential for reduction in HIV incidence in this setting due to PrEP cost [23]. In urban settings in Vietnam, daily PrEP was found to be cost-effective while event-driven PrEP was found to be cost-saving, should PrEP coverage reach 60% by 2050 among all key populations—MSM, people who inject drugs and female sex workers and their partners [24]. In Thailand, Suraratdecha and colleagues showed that providing PrEP at US$222 per person per year could be cost-effective, with an estimated ICER of US$4,836 per HIV-related disability-adjusted life year (DALY) averted when targeted at high-risk MSM or US$7,089 when targeted at all MSM over a five year period from 2017 to 2022 [17]. Without defining a cost-effectiveness threshold, the following modelling studies reported their incremental cost-effectiveness ratios (ICER). A modelling study examining PrEP implementation targeted at high-risk MSM in China estimated that PrEP could cost approximately US$24,000-US$26,000 per quality-adjusted life year gained [25]. Implementing daily PrEP coupled with twice yearly HIV testing in India was modelled to have an ICER of US$1,000 per year of life saved [26].

Considering the relatively low uptake of PrEP in Asia to date, we modelled the potential impact and cost-effectiveness of PrEP implementation and-scale up for daily as well as event-driven dosing for eight countries in Asia. To our knowledge, this is the first paper to estimate the impact and cost-effectiveness of daily versus event-driven dosing of PrEP for the entire region, including comparison of branded with generic PrEP in China.

## Methods

### Model design

This analysis was carried out using Optima HIV (v2.10.1, hiv.optimamodel.com), a population-based compartmental model [27]. Demographic, epidemiological, and behavioral estimates and data were used to inform HIV transmission among context-specific populations. Existing models for Cambodia, China, India, Indonesia, Myanmar, Nepal, Thailand, and Vietnam [28] were updated with most recent estimates for population size and HIV prevalence (S1 Table), as well as for expenditure and coverage for HIV prevention, testing, and treatment from 2010 to 2020.

### Scenario analysis

This analysis follows principles from the International Decision Support Initiative (iDSI) reference case for conducting cost-effectiveness studies [29]. To allow for consistent comparison of scenario conditions to baseline, baseline coverage of PrEP usage (% of MSM) according to AVAC was applied as of 27 January 2022: Cambodia (<500, 0%), China (4,500, 0%), India (1,500, 0%), Indonesia (<500, 0%), Myanmar (<500, 0%), Nepal (3,000, 5%), Thailand (41,000, 8%), and Vietnam (29,000, 15%) [11]. It is assumed that all existing PrEP users were MSM, though it is possible that not all users are MSM and that not all estimated users are captured in these estimates. We examined two scenarios using generic drug costs with either daily PrEP (one pill a day) or event-driven dosing (an average of 15 pills per month following the study by Molina and colleagues [30]). As a patent for branded PrEP is still in effect in China, the impact on cost-effectiveness using generic and branded TDF/FTC PrEP unit costs was examined. Latest reported HIV testing rates and antiretroviral treatment (ART) coverage as of 2021 were held constant to the end of 2026. Impact was measured through estimating the number new HIV infections and HIV-related DALYs in both the baseline and scale-up of PrEP scenarios for the entire population, as some MSM may have condomless vaginal or anal sex with cis-gender females.

#### Target population

This analysis was limited to PrEP implementation among MSM. Due to limitations in data availability across all countries, potential implementation of PrEP for transgender people was excluded. Efficacy of PrEP to prevent HIV transmission was assumed to be 82% for both daily and event-driven PrEP in the model. Efficacy has been estimated at ~99% in clinical trials [1, 30–32], the 82% efficacy value applied in this analysis corrects for lower adherence. We considered a scenario in which PrEP was implemented or scaled-up over a five-year period from the beginning of 2022 to reach coverage levels of an additional 15% of MSM by the end of 2026 compared with baseline levels, a coverage level deemed feasible by consensus of PrEP experts reached at a meeting on ‘Estimating the Cost-Effectiveness and Impact of PrEP’ held 9–10 December 2019 in Bangkok, Thailand. Within the MSM population, there are varying degrees of HIV risk. It is assumed that those engaging in the highest risk events, such as multiple sexual partners with which they engage in condomless anal sex, are also the most likely to opt-in to use PrEP as they understand their own risk profile [33]. Based on behavioural surveys with MSM in Cambodia it was estimated that 30% of MSM and transgender population accounted for 75% of higher risk acts [34]. This analysis more cautiously assumes that for all countries included, covering 15% of the highest-risk MSM will cover 30% of all commercial, regular and/or casual sexual acts among MSM. In countries with <500 of MSM currently covered by PrEP, this will mean 30% of acts among the MSM population are covered. In countries with a higher baseline coverage of PrEP such as the almost 15% of MSM currently covered in Vietnam, this may mean 60% coverage of all acts among MSM.

### Study timeframe

The timeframe for this analysis assumes new implementation or scale-up of PrEP, depending on baseline coverage, at the beginning of 2022. It assumes 15% of high-risk MSM are covered by PrEP, in addition to the baseline coverage. PrEP can be taken either with daily or event-driven dosing for five years, by the end of 2026, and coverage is assumed to stay constant thereafter. The epidemiological impact is measured by estimating HIV incidence and HIV-related DALYs from the beginning of 2022 to the end of 2031. A ten- and thirty-year timeframe are applied for impact, as it is expected that the epidemiological impact will be limited over the 5-year implementation period.

### PrEP unit costs

Costs to deliver PrEP (Table 1) to one person for one year (i.e., unit cost) for each country were derived from previous country modelling studies [17, 18, 35, 36], the WHO Global Price Report Mechanism [37], and the WHO-CHOICE project [38], and informed by expert communication with co-authors H-MS [UNAIDS], SD [WHO], and respective country partners. PrEP cost components include one HIV test session prior to PrEP initiation, antiretroviral drugs (generic TDF/FTC or TDF/3TC), health personnel time to deliver services, quarterly HIV testing, and biannual testing for sexually transmitted infections (chlamydia, syphilis, and gonorrhoea) (additional details provided in S2 Table). It is assumed that those at highest risk for acquiring HIV are identified and tested through the same mechanisms as those at lower risk. Though suggested by WHO guidelines, creatinine and hepatitis B testing were not included in these costs due to lack of data. Additionally, demand generation and effective PrEP education and promotion costs were not included due to limited data availability around the necessity of demand generation. Similarly, there was no differentiation of delivery modalities for PrEP.

**Table 1:** PrEP unit costs.

**Average estimated PrEP unit cost (USD)**				
**Country**	Daily		Event-driven*	
	Branded	Generic	Branded	Generic
Cambodia [35, 36, 38]	NA	$75	NA	$55
China **[communication with country partners through HM-S]**	$1,814	$884	$962	$504
India [36–38]	NA	$113	NA	$78
Indonesia [36, 38, 39]	NA	$321	NA	$190
Myanmar [37, 38]	NA	$118	NA	$89
Nepal [36–38, 40]	NA	$128	NA	$93
Thailand [17, 37]	NA	$252	NA	$141
Vietnam [36–38, 41]	NA	$157	NA	$112

*15 doses per month [30]

PrEP drug prices can differ substantially depending on approval and availability of generic versus branded PrEP. Communication with country partners indicated that generic PrEP is not yet registered in China, but available on the public market. The unit costs shared by the same country partners revealed that the unit cost for branded PrEP was twice as high as the unit cost for generic PrEP. As the patent is still in effect for branded PrEP in China, a cost-effectiveness analysis was conducted to compare generic and branded TDF/FTC PrEP unit costs [18]. It is uncertain as to what drives the cost of both the generic and branded PrEP in China.

### Cost per HIV-related DALY averted

To evaluate the cost-effectiveness of implementing PrEP, costs per HIV-related DALY averted were estimated for each country from 2022 to 2031, as well as the longer term 2022 to 2051. Disability weights to inform HIV-related DALYs were applied from the Global Burden of Disease study [42]. HIV-related DALYs were discounted at an annual rate of 3% until the end of 2026. ART costs saved over the 50-year period (estimated life expectancy of a PrEP user), as a result of potential new HIV infections averted through PrEP implementation, were calculated and discounted at 3% [43]. HIV-related DALYs calculated until 2031 and 2051 we also discounted at 3%. ART unit costs used to calculate the potential ART treatment costs that could be averted through PrEP administration are listed in S3 Table.

### Sensitivity analysis

A lower bound of an additional 10% and an upper bound of an additional 20% PrEP coverage, around the target of *baseline + 15% coverage*, was modelled from the beginning of 2022 to the end of 2026 to assess the impact of varying coverage levels on cost-effectiveness. To account for uncertainty relating to unit costs of PrEP, a 90% lower and a 110% upper bound for PrEP costs was also modelled.

## Results

If PrEP were implemented or scaled-up in 2022 in the eight Asian countries considered in this study, with an additional 15% coverage of MSM achieved by the end of 2026, an additional 100,000 (66,000–130,000) HIV infections (17%) and 300,000 (198,000–390,000) HIV-related DALYs (3%) could be averted over the 2022 to 2031 period compared with baseline PrEP coverage (Table 2). In addition to DALYs averted, this could result in cumulative saving of almost US$12.3 million in ART costs in all Asian countries considered.

**Table 2:** Estimated additional new HIV infections and HIV-related DALYs averted by 2031 through PrEP coverage of 15% of MSM from 2022 to 2026 (range of 10% to 20% PrEP coverage).

**Country**	**Additional new HIV infections averted**	**Percent of total HIV infections averted**	**Additional HIV-related DALYs averted**	**Percent of total HIV-related DALYs averted**
Cambodia	700 (500–900)	28% (20%-36%)	460 (300–600)	11% (8%-13%)
China	36,000 (24,300–47,400)	19% (13%-26%)	122,000 (82,000–160,300)	4% (3%-5%)
India	6,400 (4,300–8,500)	19% (13%-26%)	12,600 (8,500–16,600)	2% (2%-3%)
Indonesia	51,500 (34,300–68,800)	15% (10%-19%)	152,000 (101,700–202,800)	3% (2%-4%)
Myanmar	1,000 (700–1,400)	18% (12%-24%)	2,500 (1,700–3,300)	2% (1%-3%)
Nepal	1,000 (700–1,400)	20% (13%-26%)	3,100 (2,100–4,100)	4% (3%-5%)
Thailand	1,200 (800–1,500)	24% (16%-31%)	1,800 (1,200–2,500)	1% (1%-2%)
Vietnam	1,200 (900–1,600)	31% (21%-41%)	1,000 (700–1,300)	4% (3%-5%)

### Cost per HIV-related DALY averted

The resulting cost per HIV-related DALY averted for all scenarios are listed in Table 3. The cost per HIV-related DALYs averted was estimated to be the lowest in Indonesia, at US$600 for event-driven generic PrEP dosing at 15 days per month, if an additional 15% coverage of MSM were to be achieved by the end of 2026. This is primarily due to savings from the reduction in the unit cost and frequency of event-driven dosing of antiretrovirals (ARVs) compared with a daily regimen, as well as the estimated increasing HIV epidemic among MSM by the end of 2026. Thailand is estimated to have the highest cost per HIV-related DALY averted, while Indonesia is estimated to have the lowest cost per DALY averted. In China, event-driven generic PrEP could be more cost-effective than daily branded PrEP (Table 4).

**Table 3:** Estimated cost per HIV-related DALY averted by 2031* (USD).

**Country**	**Daily generic**	**Event-driven generic**
Cambodia	$5,500 ($4,600—$10,000)	$4,000 ($3,300—$4,600)
China	$15,600 ($13,800—$17,400)	$8,900 ($7,800—$9,900)
India	$18,100 ($16,000—$20,100)	$12,600 ($11,200—$14,100)
Indonesia	$1,000 ($900—$1,100)	$600 ($500—$700)
Myanmar	$5,300 ($4,700—$5,900)	$3,900 ($3,500– $4,400)
Nepal	$3,300 ($2,900—$3,600)	$2,400 ($2,100—$2,600)
Thailand	$34,400 ($30,300—$38,600)	$18,800 ($16,600—$21,100)
Vietnam	$15,400 ($13,200—$17,800)	$10,700 ($9,200—$12,300)

* Assuming *baseline + 15% coverage* of MSM is achieved by end of 2026 and factoring savings from ART costs averted in this timeframe and 3% discounting of costs and DALYs. Ranges defined as (10% MSM coverage and 110% PrEP unit cost) to (20% MSM coverage and 90% PrEP unit cost).

**Table 4:** Estimated cost per HIV-related DALY averted by 2031* (USD) for generic and branded PrEP in China.

**PrEP**				
**Country**	Daily branded	Daily Generic	Event-driven branded	Event-driven generic
China	$32,000 ($29,300—$34,700)	$15,600 ($14,200—$16,800)	$16,900 ($15,500—$18,400)	$8,900 ($8,100—$9,600)

*Assuming *baseline + 15% coverage* of MSM is achieved by end of 2026 and factoring savings from ART costs averted in this timeframe and 3% discounting of costs and DALYs. Ranges defined as (10% MSM coverage and 90% PrEP unit cost) to (20% MSM coverage and 110% PrEP unit cost).

To estimate the cost-effectiveness of the implementation or scale-up of PrEP in the longer term, Table 5 illustrates the cost per HIV-related DALY averted by 2051 if PrEP could reach an additional coverage of 15% of MSM in 5 years from 2022 to the end of 2026. Compared with the 2022 to 2031 period, the extra 20 years could result in a substantial reduction in the cost per HIV-related DALY. In the event-driven scenario, the cost per HIV-related DALY averted could be reduced from US$12,600 to US$4,700 (an almost 70% reduction) in India, ranging towards US$4,000 to US$400 (a 90% reduction) in Cambodia.

**Table 5:** Estimated cost per HIV-related DALY averted by 2051* (USD).

**Country**	**Daily generic**	**Event-driven generic**
Cambodia	$700 ($540—$770)	$400 ($270- $440)
China	$2,500 ($2,200—$2,800)	$1,400 ($1,200—$1,500)
India	$6,900 ($6,000—$7,700)	$4,700 ($4,200–5,300)
Indonesia	$100 ($90–110)	$100 ($90—$110)
Myanmar	$1,000 ($900—$1,100)	$700 ($630—$770)
Nepal	$500 ($450—$660)	$300 ($270—$440)
Thailand	$12,700 ($11,200—$14,300)	$6,100 ($5,400—$6,900)
Vietnam	$5,200 ($4,400—$6,200)	$3,100 ($2,600—$3,700)

*Assuming *baseline + 15% coverage* of MSM is achieved by end of 2026 and factoring savings from ART costs averted in this timeframe and 3% discounting of costs and DALYs. Ranges defined as (10% MSM coverage and 90% PrEP unit cost) to (20% MSM coverage and 110% PrEP unit cost).

## Discussion

PrEP implementation and scale-up among MSM could have a substantial public health impact in eight countries which account for over 70% of new HIV infections in Asia. If PrEP were implemented and scaled-up in 2022 in the eight Asian countries considered in this study, with an additional 15% coverage of MSM achieved by the end of 2026, an additional 100,000 (66,000–130,000) HIV infections (17%) and 300,000 (198,000–390,000) HIV-related DALYs (3%) could be averted over the 2022 to 2031 period compared with baseline PrEP coverage. In addition to HIV infections and DALYs averted, this could result in cumulative saving of almost US$12.3 million in ART costs over the five-year timeframe for these countries. Based on historical trends from 2000 onwards in the Optima HIV calibrations, it is estimated that HIV prevalence could increase among MSM in Cambodia, China, Indonesia, and Vietnam, but could decrease in India, Myanmar, and Thailand, while plateauing in Nepal, if previous trends are projected into the future. The estimates for costs per HIV-related DALY averted generally follow these trends (S2 Table). In Thailand and Vietnam, the number of new HIV infections among the entire population is projected to decrease significantly, while there is a high baseline coverage of MSM of PrEP, resulting in a higher cost per DALY averted.

Estimated costs per HIV-related DALY averted vary greatly between countries over the 2022 to 2031 period. Our results show higher costs per HIV-related DALY averted in the daily dosage scenarios compared with other studies in China [18]. However, these studies assumed higher efficacy, closer to 100%, than our assumption of 82% efficacy, which accounts for less than complete adherence. The 2022 to 2051 timeframe, however, illustrates significant reductions of 70% to 90% in the cost per HIV-related DALY averted, demonstrating the progressive cost-effectiveness of PrEP scale-up over the longer term.

The global community has yet to agree on what cost-effectiveness threshold (CET) should be applied when assessing the cost-effectiveness of an intervention [44]. Previously identified WHO-CHOICE estimates for CETs at 1–3 x GDP per capita were deemed too easily attainable in constrained low-and middle-income settings, disregarding the opportunity cost of investment in an expensive intervention for overall health [45]. The need for supply side considerations, the opportunity cost of changes in healthcare expenditure, alongside demand side considerations, the estimated social value of what an intervention ought to cost, should therefore be considered. The opportunity cost, or supply side CET, can be approximated as 0.5 x GDP per capita [46, 47]. When applying this threshold using 2020 World Bank GDP per capita estimates [48], PrEP was only demonstrated to be cost-effective in Indonesia in both the daily ($1,000 per HIV-related DALY averted by 2031) and event-driven scenarios ($600 per HIV related DALY averted by 2031) at a CET of $1,900. If the longer-term analysis to 2051 for both daily event-driven PrEP is considered with the same CET, Cambodia, China, Indonesia, Nepal are deemed cost-effective, while India, Myanmar, Thailand, and Vietnam are not. Countries in which daily and event-driven PrEP was found to be a cost-effective intervention, have increasing HIV prevalence in Optima HIV models for MSM, suggesting PrEP may be most cost-effective in settings with growing prevalence.

ARV drug costs for PrEP account for 50% to 98% of the unit costs for all countries considered, illustrating the need for countries to be able to access lower-cost PrEP. Our results show significant variation in annual drug costs for PrEP (US$38-US$1,680) across the eight countries considered. If the region could acquire PrEP drugs at the lowest cost reported in this analysis, namely US$3–4 for a bottle of 30 pills, this would greatly improve the affordability of PrEP. The comparison of daily versus event-driven PrEP use in this analysis allows for insights into the potential increased impact on cost-effectiveness. Willingness-to-pay by national governments and the ability of consumers to cover out-of-pocket expenses [49] will be a major factor in determining the feasibility of implementing and scaling PrEP, especially in countries where only branded PrEP is commonly available [50]. Nevertheless, offering event-driven PrEP as an option for MSM, in addition to daily dosing could potentially be more cost-effective, based on the results of event-driven PrEP alone. It remains critical to offer both dosing options to all potential and current PrEP users, so they may choose the method that best fits their needs.

It is unclear how the COVID-19 pandemic has influenced access to PrEP in Asia; however, recent reports suggest moderate disruption mainly due to limitations of informal access because of mobility restrictions. As an example, in Thailand there have been efforts to remedy formal access issues through telehealth consultations, laboratory sample collections at home, home delivery of drugs, and disbursement of longer supply of medication per prescription [51]. These adaptations may lower barriers to initate and adhere to PrEP for those who require it.

Study limitations include data availability and the necessity for certain assumptions to be used. Up-to-date estimates of MSM population size and HIV prevalence were not available (S1 Table). National estimates of MSM population size and HIV prevalence may also be underestimated due to stigmatization or insufficient reporting methods [52, 53] potentially resulting in an underestimation of the potential impact of PrEP. Evidence-informed assumptions needed to be made surrounding PrEP drug costs, acceptability, condomless sex surrounding PrEP use, and efficacy, including adherence to daily or event-driven dosing regimens. Efficacy was assumed to be 82% in this analysis, assuming a potentially unrealistic high adherence level of PrEP users. This analysis assumed either a daily or an event-driven scenario, though each country will have users in both scenarios, hence the actual cost per HIV-related DALY averted will lie somewhere in between the reported ranges. This analysis found higher cost per HIV-related DALY averted than other studies in the region, most likely due to this study’s conservative coverage target. If self-selection for PrEP use based on seasons of heightened risk-taking behaviour is more concentrated, as assumed in other settings [54], it may be that a higher coverage of casual acts could be achieved while PrEP may not be taken as frequently as assumed here, and PrEP cost-effectiveness would be higher as a result.

Since PrEP was introduced as part of the global HIV prevention package in 2015, its use has increased in the region [16]. However, it remains challenging to make assumptions around estimated baseline or future PrEP coverage and saturation levels, due to implementation of PrEP only being in the early stages in Asia. This study does not account for potential increased PrEP coverage through online or overseas acquisition of drugs, any medical costs to treat other sexually transmitted diseases, or additional costs for demand creation that may be necessary to increase coverage [55]. The impact and cost-effectiveness of rolling-out PrEP for people at substantial risk in other key populations and their partners needs further investigation. This includes levels of acceptability of and effective use of PrEP for additional people at substantial risk, including transgender people, sex workers, and people who inject drugs [56].

Cost-effectiveness will not be the only consideration when contemplating PrEP implementation and scale-up as reaching elimination targets may require delivery and availability of PrEP to reach those most in need, at any cost. COVID-19 has spurred further differentiation and simplification of PrEP service delivery in Asia—including offering PrEP through key population owned community clinics, distribution through primary health care, as well as through telehealth and mobile services [56, 57]. Due to enabling of refill collection and reduced stigma-related barriers, PrEP refills collected by peers or delivered by community health workers can facilitate increased demand for PrEP [7]. The addition of long-acting injectable (LAI) PrEP to the HIV prevention package could greatly optimize effective use of PrEP. Two recent LAI (cabotegravir) trials, HPTN 083 and HPTN 084, reported higher efficacy than standard oral PrEP [58, 59].

## Conclusions

Scale-up of PrEP among MSM could avert 17% new HIV infections and 3% HIV-related DALYs in eight countries in Asia over the 2022 to 2031 period compared with baseline coverage of PrEP, potentially averting US$12.3 million in ART costs and may be cost-effective in settings with rising HIV prevalence among MSM. In addition, preventing rather than treating HIV could avert lifelong medical costs and disease burden. As with all newly implemented interventions, scale-up costs for PrEP may be higher initially, but marginal cost reductions are likely to be realized with increasing economies of scale, particularly if PrEP drug costs were to be reduced. The UNAIDS 2025 targets call for robust efforts to scale-up prevention activities, including PrEP, to ensure that 95% of people at risk of HIV infection have access to and use appropriate, prioritized, person-centered, and effective combination prevention options [60]. Supplementing the combination prevention package with PrEP may reach those at risk for HIV for whom other preventative options are currently not appropriate, thereby potentially averting large numbers of HIV infections among MSM and their partners in Asia and substantially contributing to ending the HIV/AIDS epidemic.

## Supporting information

S1 Table Latest reported key epidemiological model inputs. (DOCX)

S2 Table PrEP unit cost breakdown. (DOCX)

S3 Table ART unit costs. (DOCX)
