# Supplementary material for: Cost-effectiveness and impact of pre-exposure prophylaxis to prevent HIV among men who have sex with men in Asia: A modelling study
Source: PLoS One. 2022 May 26;17(5):e0268240. doi: 10.1371/journal.pone.0268240 (PMC9135227; doi:10.1371/journal.pone.0268240)
Supplement: S1 Table — (DOCX) [file pone.0268240.s001.docx]

# Table S1.1 Latest reported key epidemiological model inputs

| Indicator | Cambodia | China | India | Indonesia | Myanmar | Nepal | Thailand | Vietnam |
| --- | --- | --- | --- | --- | --- | --- | --- | --- |
| MSM population size | 88,000 (2019) | 4,700,000† (2007) | 3,774,346* (2019) | 754,310 (2016) | 252,000 (2015) | 60,333 (2016) | 527,900 (2016) | 200,000 (2019) |
| HIV prevalence among MSM | 4.0% (2019) | 7.8% (2016) | 2.7% (2017) | 25.8% (2015) | 8.8% (2019) | 5.0% (2017) | 11.9% (2018) | 10.8% (2018) |

MSM = men who have sex with men. PrEP = pre-exposure prophylaxis.

*Estimated as 10% of the male adult population due to wide variation in MSM population size estimates

Source: Optima models (replicable with available data in these supporting documents via hiv.optimamodel.com, any queries please email info@ocds.co), 2020 UNAIDS Key Populations Atlas [1] † Wang et al [2]

REFERENCES

1. UNAIDS. UNAIDS Data 2021 Report. Geneva: UNAIDS; 2021. Available from:[<https://www.unaids.org/sites/default/files/media_asset/JC3032_AIDS_Data_book_2021_En.pdf>].

2. Wang L, Wang N Fau - Wang L, Wang L Fau - Li D, Li D Fau - Jia M, Jia M Fau - Gao X, Gao X Fau - Qu S, et al. The 2007 Estimates for People at Risk for and Living With HIV in China: Progress and Challenges. (1525-4135 (Print)).
