# Supplementary material for: Cost-effectiveness and impact of pre-exposure prophylaxis to prevent HIV among men who have sex with men in Asia: A modelling study
Source: PLoS One. 2022 May 26;17(5):e0268240. doi: 10.1371/journal.pone.0268240 (PMC9135227; doi:10.1371/journal.pone.0268240)
Supplement: S2 Table — (DOCX) [file pone.0268240.s002.docx]

# Table S1.2 PrEP unit cost breakdown

| Country | | TDF/FTC or TDF/3TC  (USD)* | HIV test  (USD)** | STI test (USD)** | Outpatient visit  (USD)** |
| --- | --- | --- | --- | --- | --- |
| Cambodia | $37.92 | $1.60 | $11.76 | $1.67 |  |
| China | $750.00 (generic) / 1,680.00 (branded) | $3.81 | $19.06 | $7.47 |  |
| India | $67.56 | $2.95 | $11.76 | $2.46 |  |
| Indonesia | $257.16 | $6.45 | $11.76 | $3.58 |  |
| Myanmar | $57.00 | $7.95 | $11.76 | $1.33 |  |
| Nepal | $68.40 | $7.95 | $11.76 | $1.08 |  |
| Thailand | $218.76 | $5.66 | $16.44 | $2.82 |  |
| Vietnam | $89.10 | $8.86 | $11.76 | $2.19 |  |

* Cost per person year based on 360 tablets per year (12 bottles of 30 tables per bottle)
** Assumed four annual HIV tests during four outpatient visits and two STI tests per year
3TC = lamivudine antiretroviral drug, FTC = emtricitabine, STI = sexually transmitted infection, TDF = tenofovir
Source: personal communication with co-authors H-MS (UNAIDS) and SD (WHO) and respective country partners
