# Supplementary material for: Cost-effectiveness and impact of pre-exposure prophylaxis to prevent HIV among men who have sex with men in Asia: A modelling study
Source: PLoS One. 2022 May 26;17(5):e0268240. doi: 10.1371/journal.pone.0268240 (PMC9135227; doi:10.1371/journal.pone.0268240)
Supplement: S3 Table — (DOCX) [file pone.0268240.s003.docx]

# Table S1.3 ART unit costs

| Country | | ART unit cost (USD)* |
| --- | --- | --- |
| Cambodia | $151.98 |  |
| China | $1,232.75 |  |
| India | $177.36 |  |
| Indonesia | $337.48 |  |
| Myanmar | $507.00 |  |
| Nepal | $547.00 |  |
| Thailand | $609.38 |  |
| Vietnam | $313.00 |  |

*Cost of delivery of ART through a health facility per person year.
Source: Optima HIV modelling studies
